# Decrease in Anti-VEGF Injections After Post-injection Endophthalmitis in Patients With Neovascular Age-Related Macular Degeneration

**DOI:** 10.1177/24741264231200470

**Published:** 2023-09-22

**Authors:** Natalia M. Binczyk, David J.A. Plemel, Mark Seamone, Christopher J. Rudnisky, Matthew T.S. Tennant

**Affiliations:** 1 Department of Ophthalmology and Visual Sciences, University of Alberta, Edmonton, AB, Canada

**Keywords:** endophthalmitis, wet AMD, anti-VEGF agents

## Abstract

**Introduction:** To evaluate the effect of antivascular endothelial growth factor (anti-VEGF)–related endophthalmitis on intravitreal injection (IVI) frequency in patients with neovascular age-related macular degeneration (nAMD). **Methods:** A retrospective chart review was performed of all cases of post IVI endophthalmitis that occurred in Edmonton, Alberta, Canada, between 2012 and 2019. Contralateral eyes affected by nAMD but without endophthalmitis served as a control group. The main outcome measures were the frequency of anti-VEGF injections, visual acuity, and activity of choroidal neovascularization before and after endophthalmitis. **Results:** Seventeen eyes met the inclusion criteria, 2 (12%) of which never resumed IVI after endophthalmitis because of the quiescence of disease. Post-endophthalmitis eyes received IVI less frequently in the 1 year after endophthalmitis (mean 0.52 ± 0.42 IVI/month) than those that received IVI 1 year before endophthalmitis (1.09 ± 0.36 IVI/month) (*P* = .001). The 17 contralateral eyes also received anti-VEGF injections less frequently after endophthalmitis than before (*P* = .001). There was no significant change in optical coherence tomography markers of disease activity in cases or controls. **Conclusions:** In patients with nAMD, endophthalmitis resolution is associated with a decrease in anti-VEGF injection frequency. The same decrease in anti-VEGF injection frequency is also seen in contralateral eyes unaffected by endophthalmitis. Markers of disease activity remain unchanged in both eyes, suggesting disease quiescence despite reduced IVI frequency.

## Introduction

Intravitreal antivascular endothelial growth factor (anti-VEGF) agents are an effective treatment for neovascular age-related macular degeneration (nAMD). Intravitreal injection (IVI) of anti-VEGF is complicated by post-injection endophthalmitis in 0.04% to 0.06% of cases.^1,2^ Previous publications identified a reduction in IVI frequency in eyes that developed endophthalmitis associated with anti-VEGF.^3–6^ The reason for the reduction in anti-VEGF frequency is not clear, although authors have hypothesized that an endophthalmitis-induced disease quiescence may be causative.^6^

The natural disease course of nAMD and the anti-VEGF injection frequency in the contralateral eye unaffected by endophthalmitis has not been studied in this patient population. The purpose of this study was to examine IVI frequency and exudative activity in patients with nAMD after anti-VEGF injection–associated endophthalmitis.

## Methods

Ethical approval for this study was obtained from the University of Alberta Research Ethics Board (Pro00097220). Participant consent was not required by the ethics board. The research adhered to the tenets of the Declaration of Helsinki.

All cases of endophthalmitis in Northern Alberta during the years 2012 to 2019 were obtained from the medical records at the Royal Alexandra Hospital in Edmonton, Alberta, Canada. Cases occurring before 2012 were excluded because the electronic operating room record system was implemented in that year. Electronic medical records (EMRs) were reviewed to include only endophthalmitis cases resulting from IVI in nAMD patients. Patients with post-injection endophthalmitis associated with diseases other than nAMD were excluded from the study.

Variables collected from the EMR included age at the time of endophthalmitis, visual acuity (VA) before and after endophthalmitis, the number and type of anti-VEGF injections before and after endophthalmitis, the type of endophthalmitis treatment, the intravitreal culture results, and spectral-domain ocular coherence tomography (OCT) (Spectralis, Heidelberg Engineering) findings including the maximum intrafluid (IRF) and subretinal fluid (SRF) height and the central foveal thickness (CFT). The data were collected for the eye affected by endophthalmitis as well as for the contralateral eye, which served as a control. Patients with nAMD and post-injection endophthalmitis but no interpretable OCT imaging at follow-up were excluded from the study.

SPSS statistical software (version 28.0.1.1, IBM) was used for data analysis. Repeated measures, such as IVI frequency, OCT markers, and Snellen VA, were analyzed using the Wilcoxon signed-rank test. The Wilcoxon rank-sum test was used to compare Snellen VA between patients who restarted IVI after endophthalmitis and those who did not. A multivariate analysis of variance and Student *t* test were used as appropriate to compare the injection frequency between eyes with endophthalmitis and controls. VA measurements of counting fingers (CF) or hand motions (HM) were converted to their Snellen equivalent using the method described by Holladay.^7^ In the involved practice, these measurements are taken at 2 ft, meaning CF is the equivalent of 20/2000 and HM is 20/20 000.^7^ Snellen VA was converted to logMAR notation for statistical analysis. In the Results section, VA is also reported with an approximate Snellen equivalent for easier readability. All data are reported as the mean ± SD. A *P* value less than 0.05 was considered statistically significant.

## Results

Twenty-eight patients with nAMD developed unilateral post-IVI endophthalmitis between the years 2012 and 2019. Five of these patients (18%) did not have interpretable follow-up OCT imaging and were thus excluded from the study. Six (26%) of the 23 contralateral eyes could not be used as controls because they did not require IVI injections and thus were excluded from the study.

Patients had been followed for 63.5 ± 43.7 months before the endophthalmitis diagnosis. Seventeen patients (65%) were women. The mean age of the patients at time of endophthalmitis was 72 ± 9 years (range, 50-87 years). The right eye was affected by endophthalmitis in 12 cases (71%). Table 1 shows the type of anti-VEGF agent used, the treatment provided, and the intravitreal culture results. Endophthalmitis was diagnosed 5.4 ± 3.3 days post-IVI, and treatment after diagnosis began at 1 ± 1.3 days.

**Table 1.** Summary of Presenting Features.

Characteristic	Cases n (%)	Controls n (%)
Anti-VEGF agent		
Aflibercept (Eylea)	4 (24)	2 (12)
Ranibizumab (Lucentis)	10 (59)	13 (76)
Bevacizumab (Avastin)	3 (18)	2 (12)
Treatment		
Intravitreal antibiotic only	3 (18)	—
Intravitreal antibiotic + PPV	14 (82)	—
Intravitreal culture results		—
*Staphylococcus epidermidis*	10 (59)	—
*Enterococcus faecalis*	1 (6)	—
*Staphylococcus aureus*	1 (6)	—
Coagulase-negative *Staphylococcus*	2 (12)	—
*Staphylococcus lugdunensis*	1 (6)	—
*Micrococcus luteus* (possible contaminant)	1 (6)	—
No growth	1 (6)	—

Abbreviations: Anti-VEGF, antivascular endothelial growth factor; PPV, pars plana vitrectomy.

All 17 patients maintained follow-up for at least 12 months, with a mean follow-up of 39.1 ± 24.3 months. Two eyes (12%) never resumed IVI after developing endophthalmitis. Twelve eyes resumed anti-VEGF injections in the first year after endophthalmitis. Anti-VEGF was resumed 44 ± 30 days after endophthalmitis. In those who restarted anti-VEGF, the VA was 0.44 ± 0.28 logMAR (Snellen equivalent 20/50) before endophthalmitis and 0.75 ± 0.54 logMAR (Snellen equivalent 20/114) after endophthalmitis (*P* = .023). In those who did not restart anti-VEGF, the VA was 0.52 ± 0.22 logMAR (Snellen equivalent 20/70) and 0.51 ± 0.22 logMAR (Snellen equivalent 20/63, respectively (*P* = .5). Table 2 shows the proportions of patients on IVI after endophthalmitis at a given time.

**Table 2.** Follow-up by Treatment Status (Initial Sample = 17).

	Number (%)		
Follow-up	NoTreatment Necessary^a^	Resumed Treatment	Total Affected Eyes
>3 months	7 (41)	10 (59)	17 (100)
>6 months	7 (41)	10 (59)	17 (100)
>12 months	6 (35)	11 (65)	17 (100)
>24 months	3 (18)	8 (47)	11 (65)
>36 months	3 (18)	5 (29)	8 (47)
>48 months	2 (12)	4 (23)	6 (35)

a After endophthalmitis.

**Figure 1.**
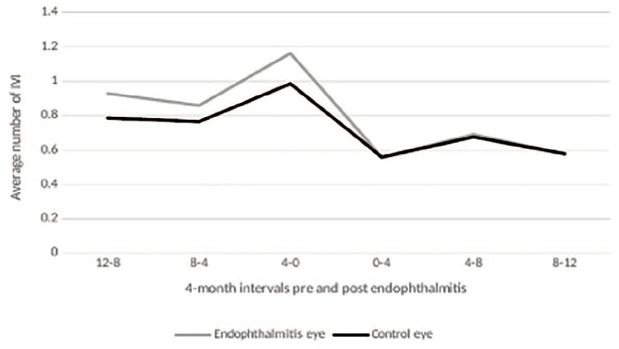
Mean monthly intravitreal injections (IVIs). Data were split into 4-month blocks to show the trend over time (sample size = 17).

Injection frequency decreased in the year after endophthalmitis compared with the year preceding endophthalmitis in both cases and controls (Table 3). The injection burden was not statistically different between eyes with endophthalmitis and control eyes before endophthalmitis (*P* = .37). Figure 1 shows the injection frequency split into 4-month time periods to show the trend over time. Of the 17 patients with endophthalmitis, 16 (94%) were culture positive. The injection frequency after endophthalmitis was not associated with culture results. In the first year after endophthalmitis, the monthly IVI frequency was 0.69 ± 0.33 for those who were culture positive and 0.79 ± 0.14 for the patient who was culture negative.

**Table 3.** Injection Frequency 1 Year Before and 1 Year After Endophthalmitis.

		Mean Injections/Month ± SD		
Group	n	Before Endophthalmitis	After Endophthalmitis	P Value
Cases	17	1.09 ± 0.36	0.52 ± 0.42	<.001
Controls	17	0.95 ± 0.51	0.58 ± 0.41	<.001

Markers of nAMD disease, including the CFT, maximum IRF and SRF, and percentage of dry retinas seen on OCT, were analyzed in both eyes that developed endophthalmitis (Table 4) and their fellow control eyes (Table 5). There was no change in the markers of nAMD disease activity in either eye (*P* > .05). The VA was reduced in the eyes with endophthalmitis (*P* < .019; Table 4) but not in control eyes (*P* = .877; Table 5).

**Table 4.** Visual Acuity, Central Foveal Thickness, Maximum Intraretinal and Subretinal Fluid, and Percentage of Dry Retinas at All Timepoints (Sample = 17).

	Visual Acuity				
Timepoint	Mean logMAR ± SD	Snellen Equivalent	Mean CFT (μm) ± SD	Mean Max IRF/SRF (μm) ± SD	Mean % of Dry Retinas ± SD
Pre-endophthalmitis	−0.47 ± 0.26	20/50	306 ± 67	50 ± 50	56 ± 38
Post-endophthalmitis	−0.68 ± 1.42	20/200	301 ± 112	64 ± 97	52 ± 46
*P* value^a^	.019		.836	.877	.756

Abbreviations: CFT, central foveal thickness; IRF, intrafluid; SRF, subretinal fluid.

a Pre-endophthalmitis vs post-endophthalmitis (Wilcoxon signed-rank test).

**Table 5.** Visual Acuity, Central Foveal Thickness, Maximum Intraretinal and Subretinal Fluid, and Percentage of Dry Retinas in Control Group at All Timepoints (Sample = 17).

	Visual Acuity				
Timepoint	Mean logMAR ± SD	Snellen Equivalent	Mean CFT (μm) ± SD	Mean Max IRF/SRF (μm) ± SD	Mean % of Dry Retinas ± SD
Pre-endophthalmitis	−0.53 ± 0.41	20/70	334 ± 149	62 ± 103	55 ± 37
Post-endophthalmitis	−0.09 ± 1.86	20/70	322 ± 201	64 ± 155	61 ± 43
*P* value^a^	.877		.332	.776	.308

Abbreviations: CFT, central foveal thickness; IRF, intrafluid; SRF, subretinal fluid.

a Pre-endophthalmitis vs post-endophthalmitis (Wilcoxon signed-rank test).

## Conclusions

Numerous reports have shown a reduction in IVI frequency after endophthalmitis. Some studies suggest that the decrease in IVI frequency is the result of endophthalmitis-induced quiescence of exudative activity. Our study adds to the literature by showing what occurs in the contralateral eye that also had nAMD but was unaffected by endophthalmitis. This retrospective analysis of IVI-associated endophthalmitis in nAMD showed that both affected eyes and control eyes had a decrease in injection frequency over time with concurrent anatomic signs of disease stability.

Emoto et al^4^ first suggested there may be an endophthalmitis-induced regression of choroidal neovascularization after vitrectomy for IVI-associated endophthalmitis in a series of 2 patients. The 2 culture-negative endophthalmitis cases resulted in geographic atrophy but no resumption of anti-VEGF. Kally et al^5^ described a case in which an eye with nAMD that developed IVI-associated coagulase-negative staphylococci endophthalmitis required no further anti-VEGF after infection management. Kokame et al^3^ described a series of 7 eyes with nAMD and IVI-associated endophthalmitis; 6 eyes needed no further anti-VEGF after treatment for infection. These publications observed a decrease in SRF after resolution of endophthalmitis.

After the aforementioned studies, Arnett et al^6^ questioned whether quiescence of exudative maculopathy or low vision from endophthalmitis sequelae was the basis for the cessation of anti-VEGF treatment. In their series, eyes that lost 2 or more lines of vision from endophthalmitis were excluded. Of the 21 eyes included, 7 needed no further IVI after endophthalmitis and those that did resume IVI did so at a reduced frequency with signs of anatomic disease stability on OCT. Arnett et al concluded that, independent of VA loss, endophthalmitis resolution is associated with a reduction in IVI frequency and a decrease in choroidal exudative activity.

Our study adds to these earlier publications. If Arnett et al^6^ showed that the reduction in IVI frequency is not the result of clinicians treating nAMD less aggressively because of a reduction in VA, we have added that the reduction in IVI frequency is not unique to the eye with endophthalmitis. The decrease in anti-VEGF frequency after endophthalmitis may be caused by 1 or more factors. The decrease in IVI frequency could be the result of patient or physician hesitancy to treat after endophthalmitis, the natural history of nAMD in which fewer injections are needed over time, or another factor. This study shows that both the affected eye and control eye have a reduction in anti-VEGF requirements after endophthalmitis. It is unclear what underlies that effect.

Endophthalmitis can be treated with intravitreal antibiotics, pars plana vitrectomy (PPV), or both. It was previously shown that anti-VEGF agents have a shorter half-life in vitrectomized eyes.^8–10^ Therefore, it would be expected that eyes would require more frequent IVI for treatment of nAMD after PPV. The reverse has been found in case series showing reduced IVI frequency after endophthalmitis vitrectomy treatment.^3–6^

Theories have been proposed as to why endophthalmitis would alter nAMD activity. Early reports suggested that vitrectomy, by lowering intraocular VEGF levels and the removal of chronic traction on the macula, was the cause of disease quiescence.^4^ This theory was challenged in a later series that showed similar disease regression despite most eyes receiving intravitreal antibiotics only, without PPV, for the treatment of anti-VEGF–associated endophthalmitis.^3^ An anti-angiogenic effect from the infection, through upregulation of complement factor H or upregulation of gunylate-binding protein, has been proposed for post-endophthalmitis eyes.^3,5^ Others have suggested that inflammation, rather than the infection, induces a relative involution of nAMD. A series of eyes that developed noninfectious endophthalmitis after intravitreal triamcinolone for cystoid macular edema showed a reduction in disease activity with observation only.^11^ The mean foveal thickness and VA improved after sterile inflammation. Alternatively, endophthalmitis does not change exudative activity in eyes with AMD and the decrease in IVI frequency is the result of other factors.

There are limitations to our study. Because of its retrospective nature, patients had variable follow-up and were reported differently. Patients without interpretable OCT findings were excluded from the study because it was not possible to determine whether a decrease in IVI frequency was associated with disease quiescence. Different treatment patterns, such as scheduled or treat-and-extend, might lead physicians to limit treatment after endophthalmitis. Physicians or patients could be hesitant to continue treatment after a serious complication occurs, thereby reducing IVI frequency.

Our study found that endophthalmitis associated with intravitreal anti-VEGF resulted in a reduced frequency of IVI in eyes with endophthalmitis and control eyes. Signs of exudative maculopathy were stable, despite fewer treatments. Neither culture results nor VA after endophthalmitis influenced post-endophthalmitis IVI frequency. The reduced frequency of IVI after endophthalmitis in nAMD eyes may be the result of the natural disease course wherein eyes require fewer anti-VEGF over time, hesitancy to treat as aggressively after endophthalmitis, or other factors.
